# Supplementary material for: Cycloguanil and Analogues Potently Target DHFR in Cancer Cells to Elicit Anti-Cancer Activity
Source: Metabolites. 2023 Jan 19;13(2):151. doi: 10.3390/metabo13020151 (PMC9961069; doi:10.3390/metabo13020151)
Supplement: Supplementary file 1 [file metabolites-13-00151-s001.zip › metabolites-2132984-supplementary.pdf]

Supplementary Materials for:

Cycloguanil and analogues target DHFR in cancer cells to elicit anti-cancer activity

Jennifer I. Brown [1], Peng Wang [2,3], Alan Y. L. Wong [2,4], Boryana Petrova [2,3], Rosanne Persaud [1], Sepideh Soukhtehzari [1], Melanie Lopez McDonald [5], Danielle Hanke [1], Josephine Christensen [1], Petar Iliev [1], Weiyuan Wang [5], Daniel K. Everton [1], Karla C. Williams [1], David A. Frank [5], Naama Kanarek [2,3,6], and Brent D. G. Page [1,*]

[1] Faculty of Pharmaceutical Sciences, University of British Columbia, Vancouver, BC, Canada
[2] Department of Pathology, Boston Children's Hospital, Boston, MA, 02115,USA
[3] Harvard Medical School, Boston, MA, 02115, USA
[4] Harvard MD-PhD Program, Harvard Medical School, Boston, MA, 02115, USA
[5] Department of Hematology and Medical Oncology, Emory University, Atlanta, GA, USA
[6] The Broad Institute of Harvard and MIT, Cambridge, MA, USA
[*] Correspondence: brent.page@ubc.ca

List of Figures

Figure S1. Ligand-receptor 2D interaction diagrams for the top pose of each docked compound 2
Figure S2. Cycloguanil analogues inhibit DHFR enzymatic activity *in vitro* 4
Figure S3. Linear range of DHFR enzymatic assay 5
Figure S4. Melt curve for DHFR in cell lysate. 8
Figure S5. Uncropped Western blots for data presented in Figure S4 9
Figure S6. Uncropped Western blots for data presented in Figure 5a and Figure 5b 11
Figure S7. Uncropped Western blots for data presented in Figure 5c 12
Figure S8. Uncropped Western blots for data presented in Figure 5d 13
Figure S9. Impact of cycloguanil analogues on thymidine phosphorylate thermal stability. 14
Figure S10. Uncropped Western blots for data presented in Figure S9a 15
Figure S11. Impact of cycloguanil analogues on wound healing. 16
Figure S12. Folinic acid supplementation significantly increases folate species that are downstream of DHFR. 17
Figure S13. DHFR inhibitors block STAT3-dependent gene expression. 20

List of Tables

Table S1. Number of References found by structure search using SciFinder^n as of January, 2023 3
Table S2. Comparison of GI_{50} values for each inhibitor from Human Tumor Cell Lines Screen reveal significant differences 5
Table S3. GI_{50} values for each inhibitor compared to the methotrexate gold standard separated by cell line 6
Table S4. Cell viability with inhibitor alone and together with folinic acid 7
Table S5. CETSA analysis of DHFR in lysate treated with cycloguanil-like triazene compounds 10
Table S6. Statistical comparisons for the data presented in Figure 6 18
Table S7. Statistical comparisons for the data presented in Figure 7 and Figure S13 21

Supplementary Methods 22
References 22

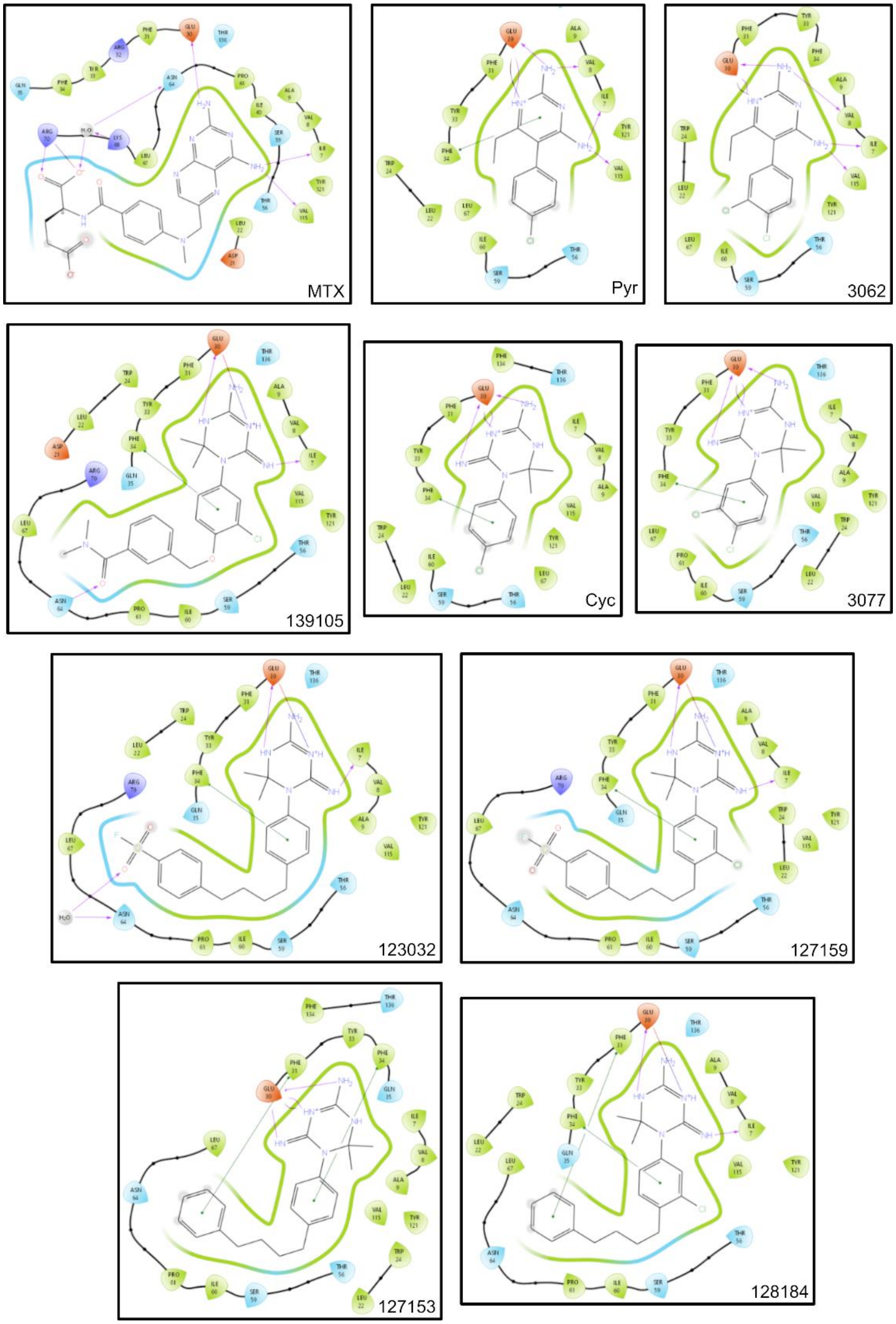

Figure S1. Ligand-receptor 2D interaction diagrams for the top pose of each docked compound. Diagrams highlight the conserved hydrogen bonding (purple arrow) between the heterocycles of each compound and Glu30 of the DHFR folate-binding site. Further conserved pi-stacking (green line) between the phenzyl moiety and Phe34 of most compounds is also highlighted. Orange residues are negatively charged, green residues are hydrophobic, and blue residues are polar. Ligand-receptor interactions were visualized using Maestro (Schrödinger).

Table S1. Number of References found by structure search using SciFinder^{n} as of January, 2023.

**Compound**	**Number of References as of Jan. 2023**		
**NSC Number**	**Neutral**	**HCl Salt**	**$EtSO_3$ Salt**
**123032**	9	0	1
**127159**	6	0	1
**127153**	11	2	0
**128184**	8	1	0

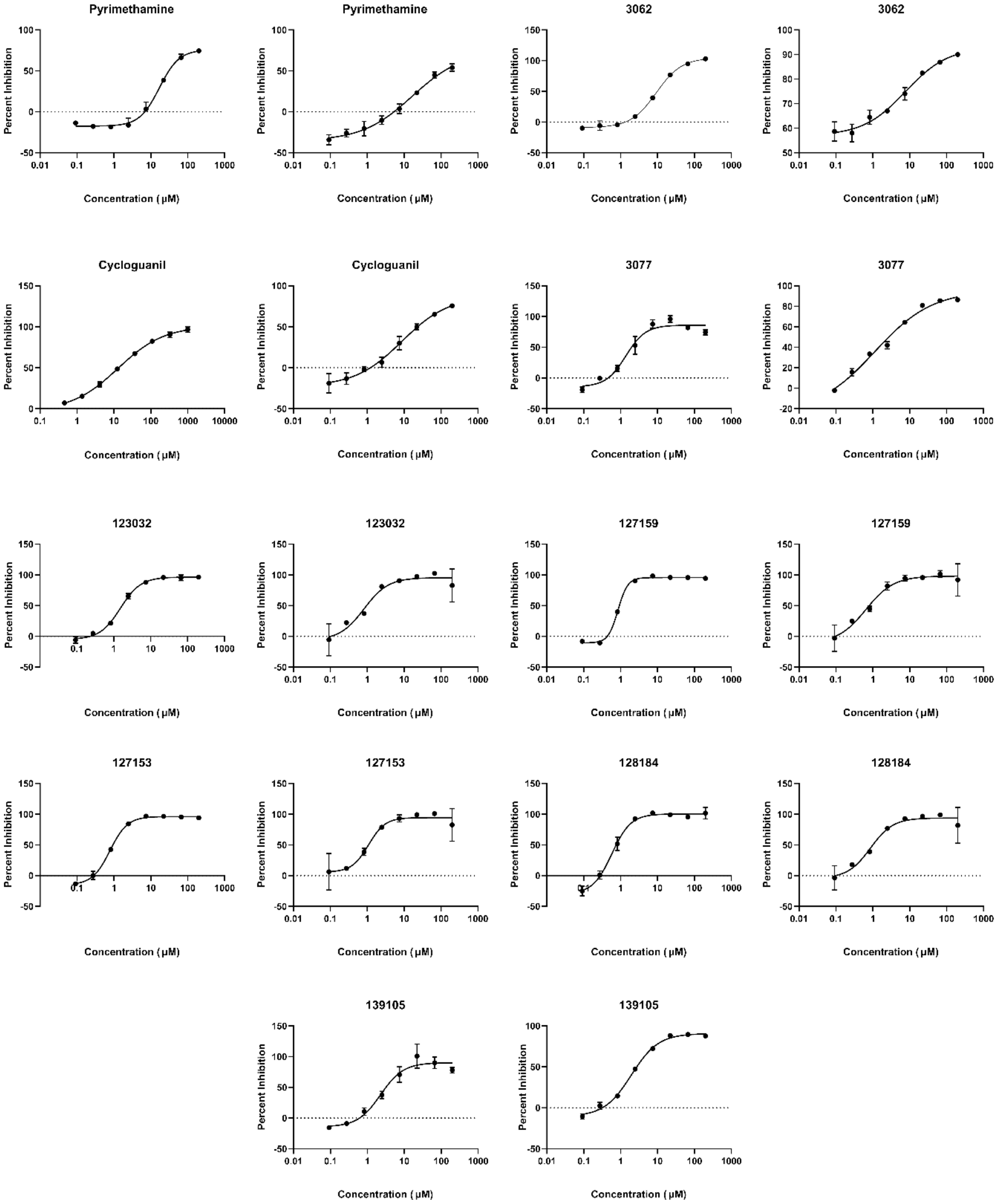

Figure S2. Cycloguanil analogues inhibit DHFR enzymatic activity *in vitro*. An enzymatic activity assay was performed for cycloguanil analogues and pyrimethamine and its chlorinated analogue 3062 by monitoring absorbance of NADPH at 340 nm. Percent inhibition was determined from absorbance values using equation (1). Percent inhibition was plotted against the concentration of each inhibitor (µM, Log_{10} scale) and IC_{50} values were determined by fitting to equation (2) using GraphPad Prism Version 9.0. Experiments were performed in duplicate and two independent experiments were performed (n = 2). Curves for *in vitro* inhibition of DHFR by methotrexate can be found in Heppler et al., 2022 [1].

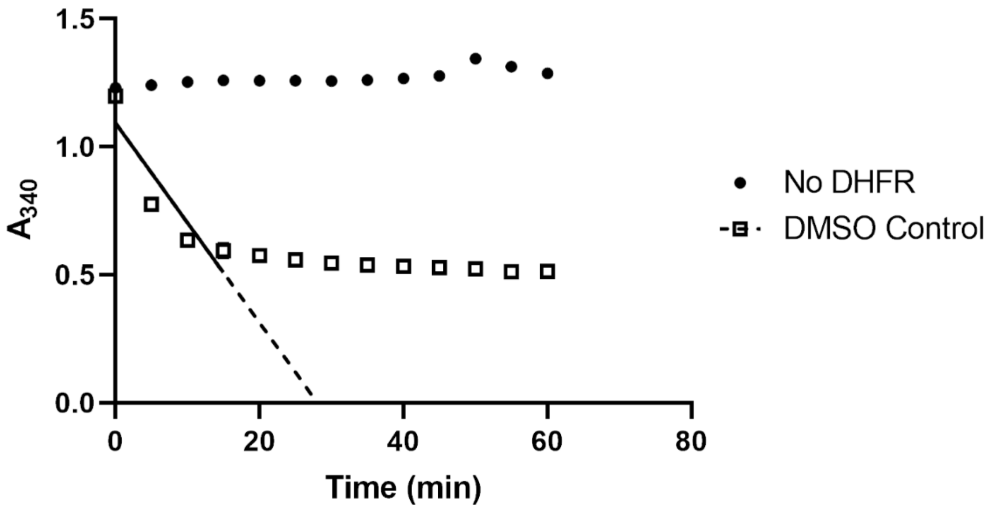

Figure S3. Linear range of DHFR enzymatic assay. A representative plot of No DHFR (black circles) and DMSO control (open square) reactions over 60 min. In the DMSO control representing 100% DHFR enzyme activity, DHFR consumption of NADPH is linear within 15 min of reaction initiation (dashed line of best fit $R^2 = 0.8295$). Further, degradation of NADPH in the No DHFR control is negligible over 60 min. Error bars represent the standard deviation (n=2), but most are too tight to be visible.

Table S2. Comparison of GI_{50} values for each inhibitor from Human Tumor Cell Lines Screen reveal significant differences. *p* values of each comparison of GI_{50} Pearson Correlation coefficients are shown. Resulting correlations of each comparison are shown in **Figure 2a**. Each comparison is significantly different, as determined by a two-tailed statistical analysis.

	**MTX**	**Pyr**	**3062**	**Cyc**	**3077**	**123032**	**127159**	**128184**	**139105**
**MTX**		1.02×10^{-8}	1.98×10^{-9}	0.0050	7.80×10^{-8}	9.00×10^{-5}	1.61×10^{-6}	4.00×10^{-5}	1.32×10^{-10}
**Pyr**	1.02×10^{-8}		1.69×10^{-8}	0.0050	4.58×10^{-6}	5.00×10^{-5}	5.05×10^{-5}	1.00×10^{-5}	4.00×10^{-6}
**3062**	1.98×10^{-9}	1.69×10^{-8}		0.0030	0.0011	0.043	0.0055	0.0066	0.0011
**Cyc**	0.0050	0.0050	0.0030		6.31×10^{-10}	0.00019	6.40×10^{-5}	0.00038	0.015
**3077**	7.80×10^{-8}	4.58×10^{-6}	0.0011	6.31×10^{-10}		1.14×10^{-6}	1.77×10^{-7}	1.96×10^{-9}	5.46×10^{-8}
**123032**	9.00×10^{-5}	5.00×10^{-5}	0.043	0.00019	1.14×10^{-6}		1.97×10^{-23}	1.90×10^{-14}	1.42×10^{-12}
**127159**	1.61×10^{-6}	5.05×10^{-5}	0.0055	6.40×10^{-5}	1.77×10^{-7}	1.97×10^{-23}		8.35×10^{-13}	4.07×10^{-12}
**128184**	4.00×10^{-5}	1.00×10^{-5}	0.0066	0.00038	1.96×10^{-9}	1.90×10^{-14}	8.35×10^{-13}		3.91×10^{-12}
**139105**	1.32×10^{-10}	4.00×10^{-6}	0.0011	0.015	5.46×10^{-8}	1.42×10^{-12}	4.07×10^{-12}	3.91×10^{-12}	

Table S3. GI_{50} values for each inhibitor compared to the methotrexate gold standard separated by cell line. *p* values of each comparison of GI_{50} Pearson Correlation coefficients are shown. Resulting correlations of each comparison are shown in **Figure 2b**. These data reveal that each inhibitor has significant correlations to methotrexate for both melanoma and breast cancer cell types. Statistical significance was determined by a two-tailed statistical analysis.

	Leukemia	Non-Small Cell Lung	Colon	CNS	Melanoma	Ovarian	Renal	Breast
**Pyr**	0.0332	0.0898	0.0298	0.0688	0.0048	0.0472	0.1498	0.2974
**3062**		0.0028	0.2236	0.0665	0.0081	0.0399	0.0791	0.1469
**Cyc**	0.5296	0.6265	0.1959	0.1212	0.0077	0.1361	0.4639	0.6564
**3077**	0.2265	0.0126	0.0732	0.0045	0.0029	0.627	0.0743	0.0338
**123032**	0.8031	0.2505	0.2349	0.5109	0.0041	0.5011	0.5997	0.0087
**127159**	0.4798	0.1421	0.3722	0.3256	0.0103	0.4439	0.8261	0.0108
**128184**	0.1644	0.0015	0.917	0.4146	0.0136	0.9486	0.9731	0.0193
**139105**	0.0890	0.0012	0.6835	0.0016	0.0001	0.3423	0.3574	0.007

Table S4. Cell viability with inhibitor alone and together with folinic acid. To determine statistical significance between each treatment with and without folinic acid, data were analyzed using an Ordinary one-way ANOVA with the Šidák method for multiple comparisons. Error represents standard deviation of three replicates from three independent experiments (n=3).

Treatment	Relative Viability (%) – Inhibitor Alone	Relative Viability (%) – Inhibitor + FA Treatment	Adjusted *p* value (compared to Inhibitor Alone treatment)	Significance
MCF-7				
DMSO	100.00 ± 0.0	108.19 ± 4.1	0.9647	ns
Pyrimethamine	85.23 ± 3.6	94.91 ± 5.5	0.8965	ns
NSC3062	65.16 ± 9.0	100.10 ± 8.2	0.0002	***
Methotrexate	68.18 ± 8.9	108.50 ± 11.7	<0.0001	****
Cycloguanil	87.69 ± 3.1	105.36 ± 5.9	0.1832	ns
NSC3077	68.81 ± 6.9	98.96 ± 0.6	0.0015	**
NSC123032	81.94 ± 19.4	104.16 ± 6.3	0.0381	*
NSC127159	67.68 ± 13.3	99.99 ± 3.2	0.0006	***
NSC127153	43.03 ± 13.9	56.75 ± 7.9	0.5132	ns
NSC128184	35.94 ± 11.4	48.65 ± 6.9	0.6216	ns
NSC139105	66.53 ± 8.1	82.83 ± 9.7	0.2738	ns
MDA-MB-231				
DMSO	100.00 ± 0.0	108.64 ± 10.1	0.9968	ns
Pyrimethamine	55.66 ± 9.9	102.92 ± 19.8	0.0004	***
NSC3062	52.76 ± 12.1	65.52 ± 15.0	0.9380	ns
Methotrexate	53.86 ± 10.7	95.03 ± 17.6	0.0027	**
Cycloguanil	57.04 ± 11.0	87.67 ± 20.5	0.0522	ns
NSC3077	57.38 ± 10.0	66.76 ± 10.4	0.9937	ns
NSC123032	57.55 ± 3.1	103.45 ± 16.5	0.0006	***
NSC127159	50.08 ± 8.8	106.42 ± 15.0	<0.0001	****
NSC127153	45.99 ± 10.6	55.71 ± 12.7	0.9915	ns
NSC128184	42.94 ± 9.7	53.14 ± 12.7	0.9875	ns
NSC139105	46.35 ± 9.0	65.85 ± 13.7	0.5260	ns
MDA-MB-468				
DMSO	100.00 ± 0.0	120.77 ± 9.7	0.0564	ns
Pyrimethamine	75.94 ± 2.0	97.20 ± 16.4	0.0472	*
NSC3062	65.38 ± 1.3	81.08 ± 13.2	0.2980	ns
Methotrexate	64.00 ± 5.5	81.09 ± 6.9	0.1986	ns
Cycloguanil	76.08 ± 10.5	84.36 ± 3.0	0.9566	ns
NSC3077	67.95 ± 6.1	69.82 ± 1.0	>0.9999	ns
NSC123032	71.41 ± 10.0	66.59 ± 2.1	0.9995	ns
NSC127159	70.45 ± 7.8	77.23 ± 7.2	0.9902	ns
NSC127153	17.90 ± 7.8	23.92 ± 7.7	0.9963	ns
NSC128184	12.35 ± 8.6	16.09 ± 10.4	>0.9999	ns
NSC139105	57.09 ± 8.0	81.38 ± 16.9	0.0143	*

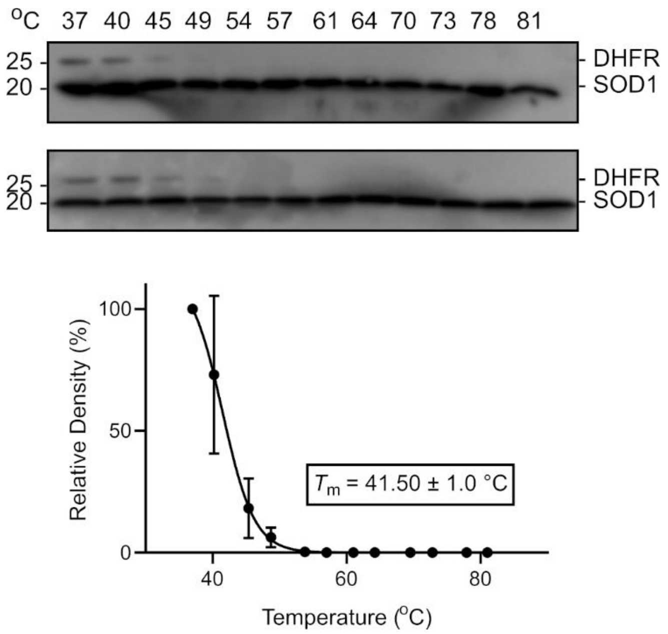

Figure S4. Melt curve for DHFR in cell lysate. MDA-MB-468 lysates were incubated from 37 to 81°C for 3 min. The soluble fraction was separated from insoluble, denatured proteins by centrifugation and separated via SDS-PAGE. The density of the DHFR band (~21 kDa) was normalized to the SOD1 band (~15 kDa) in each lane and subsequently normalized to 37°C as 100% (top). Relative densities were plotted against temperature and data were fit to equation (3) to determine the melting temperature (T_m) (bottom). All control reactions were performed in duplicate. Error bars represent the standard deviation.

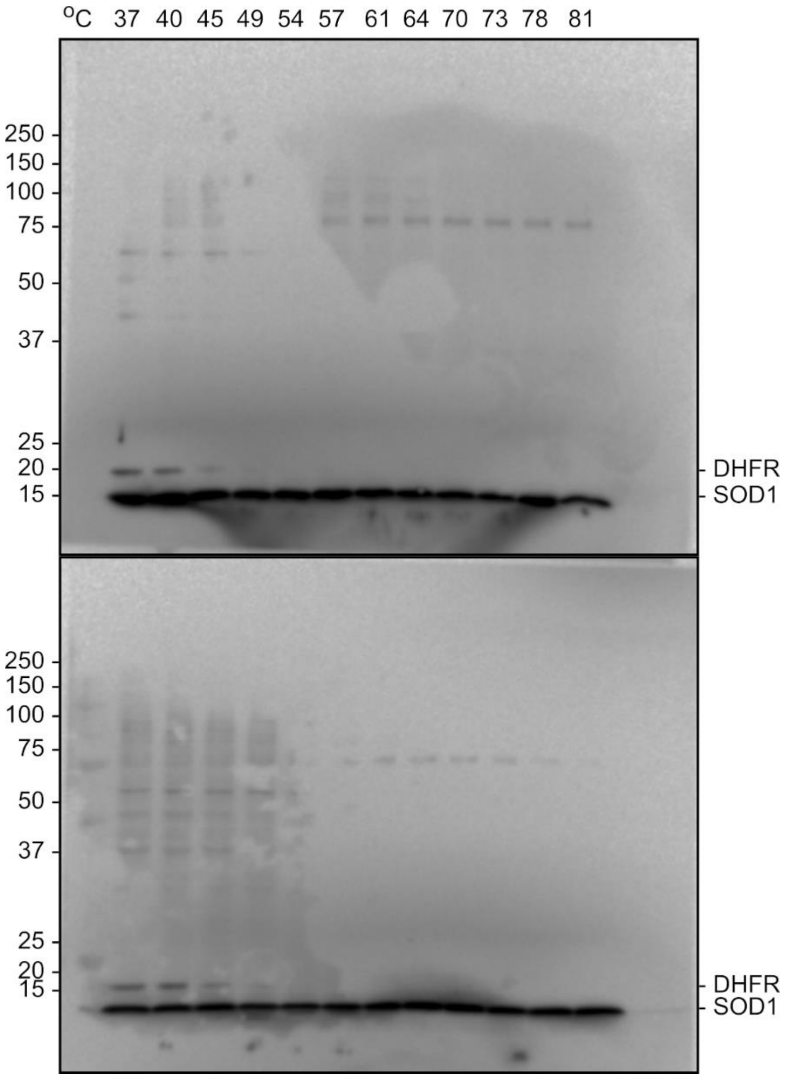

Figure S5. Uncropped Western blots for data presented in **Figure S4**. MDA-MB-468 lysates were incubated from 37 to 81°C for 3 min. The soluble fraction was separated from insoluble, denatured proteins by centrifugation and separated via SDS-PAGE. anti-DHFR (from mouse) was used at a dilution of 1:200, anti-SOD1 (from mouse) was used at a dilution of 1:500, and HRP-linked anti-mouse IgG was used at a concentration of 1:3000.

Table S5. CETSA analysis of DHFR in lysate treated with cycloguanil-like triazene compounds. To determine statistical significance between each treatment and the DMSO-control, data were analyzed using an Ordinary one-way ANOVA with Dunnett's multiple comparisons. Error represents standard deviation of five replicates from three independent experiments (n=5) (except for NSC3062, standard deviation of four replicates from two independent experiments (n=4)).

Treatment	Temperature (°C)	Normalized Band Density (%)	Adjusted *p* value (compared to DMSO control at 45°C)	Significance
DMSO	37	100.00 ± 0.0	0.0152	*
DMSO	45	20.62 ± 0.6	-	-
Pyrimethamine	45	104.12 ± 33.5	0.0095	**
NSC3062	45	83.56 ± 52.1	0.1186	ns
Methotrexate	45	123.37 ± 73.5	0.0009	***
Cycloguanil	45	125.67 ± 39.6	0.0007	***
NSC3077	45	106.38 ± 29.5	0.0072	**
NSC123032	45	121.24 ± 45.6	0.0011	**
NSC127159	45	99.96 ± 26.2	0.0153	*
NSC127153	45	100.16 ± 36.4	0.0150	*
NSC128184	45	92. 65 ± 34.6	0.0341	*
NSC139105	45	86.05 ± 21.9	0.0669	ns

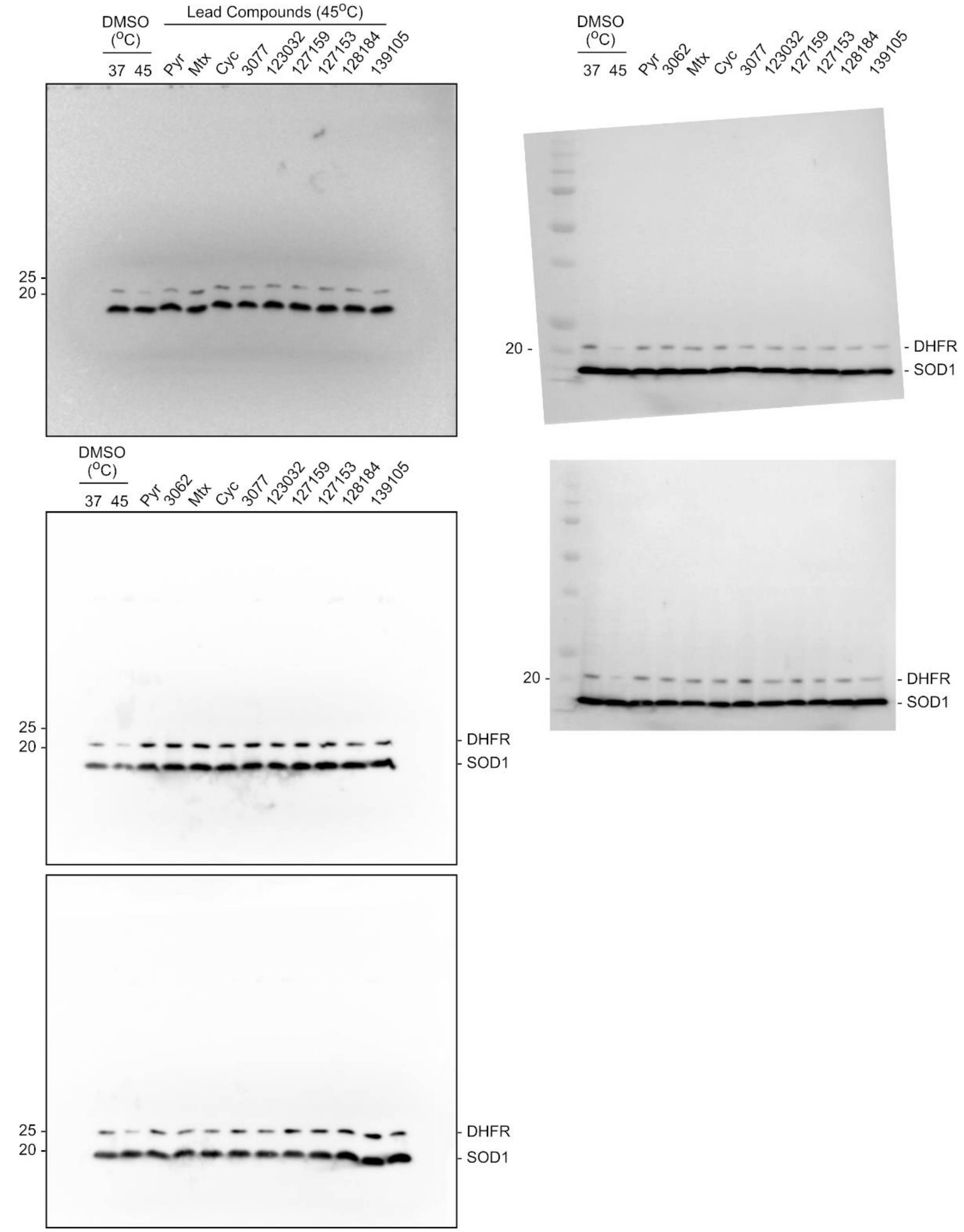

Figure S6. Uncropped Western blots for data presented in **Figure 5a** and **Figure 5b**. MDA-MB-468 lysates were treated with 10 μM each compound and incubated at 45°C. Lysates were electrophoretically separated and DHFR was detected *via* Western blot. anti-DHFR (from mouse) was used at a dilution of 1:200, anti-SOD1 (from mouse) was used at a dilution of 1:500, and HRP-linked anti-mouse IgG was used at a concentration of 1:3000.

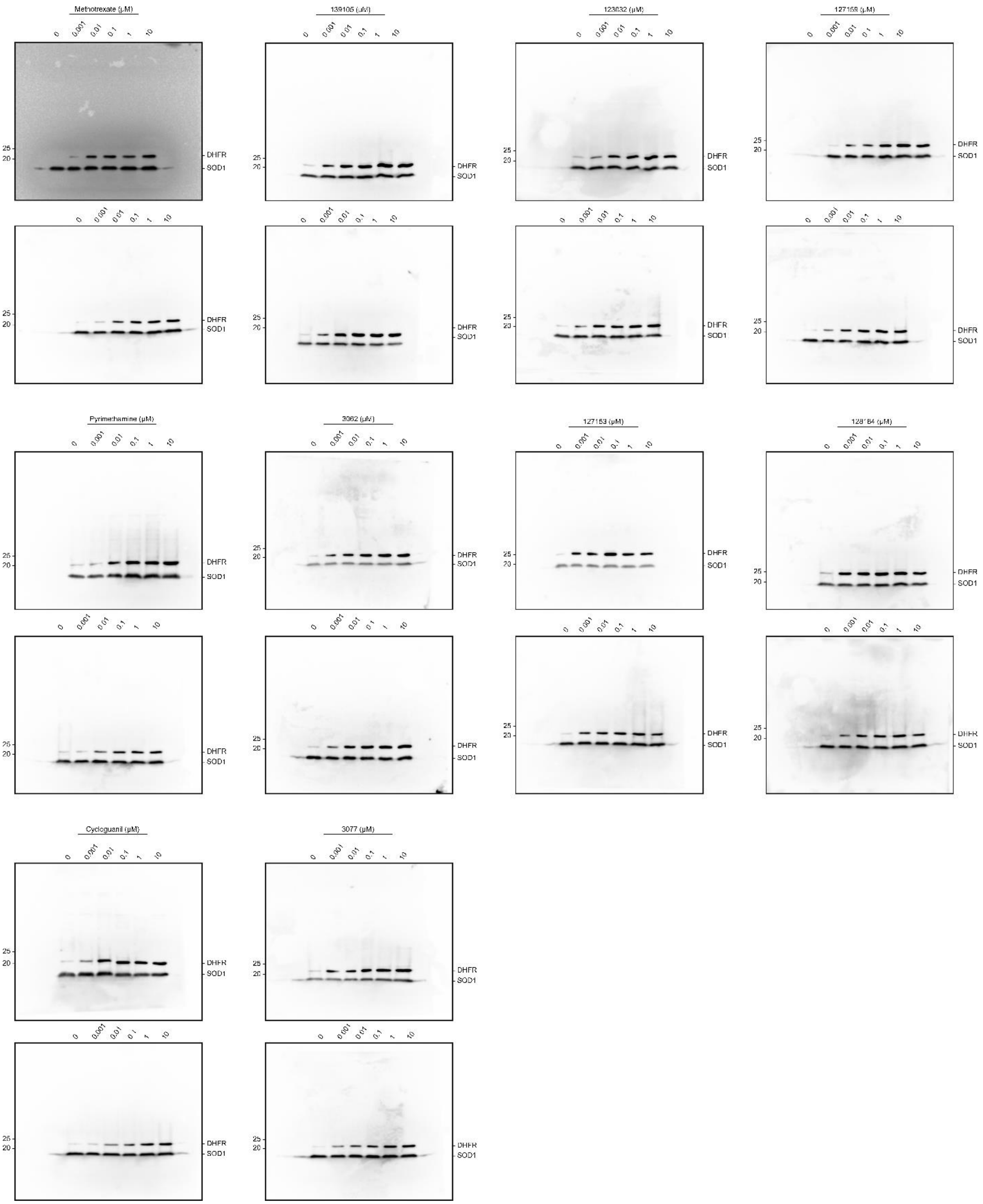

Figure S7. Uncropped Western blots for data presented in **Figure 5c**. MDA-MB-468 cells were treated with 0 – 10 µM of each inhibitor for 24 h. DHFR and the thermostable SOD1 were detected *via* Western blot. anti-DHFR (from mouse) was used at a dilution of 1:200, anti-SOD1 (from mouse) was used at a dilution of 1:500, and HRP-linked anti-mouse IgG was used at a concentration of 1:3000.

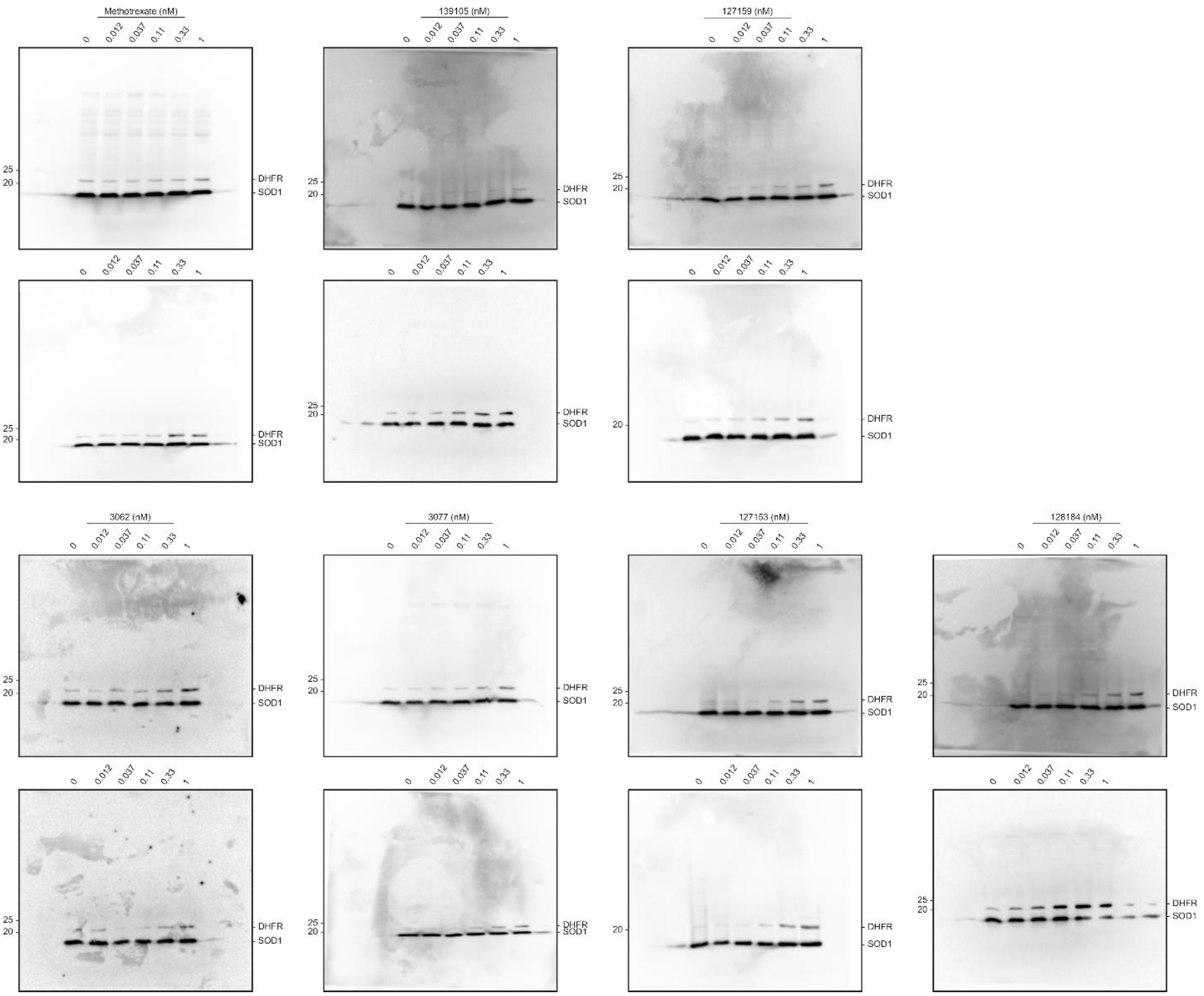

Figure S8. Uncropped Western blots for data presented in **Figure 5d**. MDA-MB-468 cells were treated with 0 – 1 nM of each inhibitor for 24 h. DHFR and the thermostable SOD1 were detected *via* Western blot. anti-DHFR (from mouse) was used at a dilution of 1:200, anti-SOD1 (from mouse) was used at a dilution of 1:500, and HRP-linked anti-mouse IgG was used at a concentration of 1:3000.

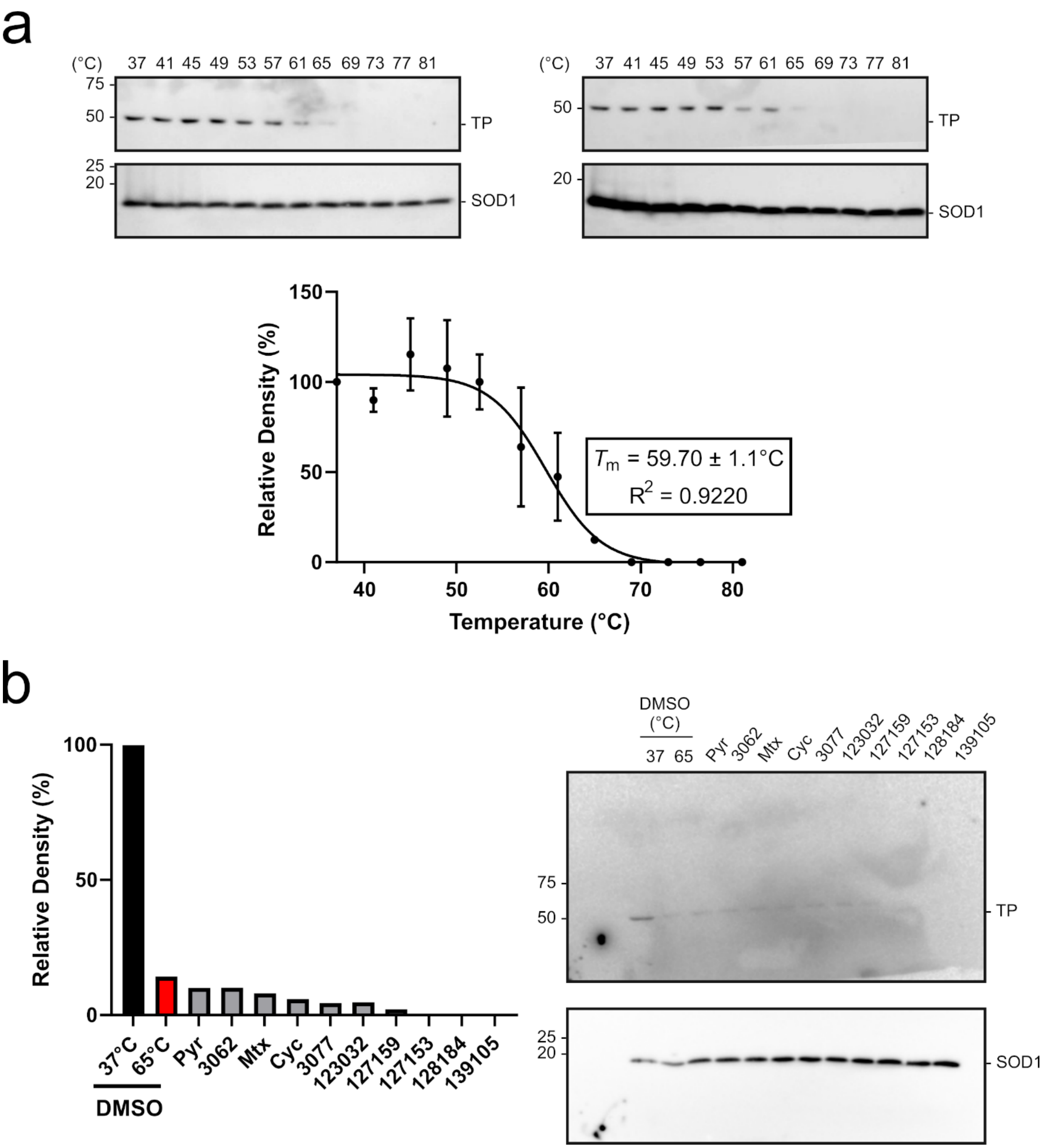

Figure S9. Impact of cycloguanil analogues on thymidine phosphorylate thermal stability. (a) Melt curve for TP in cell lysate. MDA-MB-468 lysates were incubated from 37 to 81°C for 3 min. The soluble fraction was separated from insoluble, denatured proteins by centrifugation and separated via SDS-PAGE. The density of the TP band (~50 kDa) was normalized to the SOD1 band (~15 kDa) in each lane and subsequently normalized to 37°C as 100% (top). Relative densities were plotted against temperature and data were fit to equation (3) to determine the melting temperature (T_m) (bottom). Reactions were performed in duplicate. Error bars represent the standard deviation. (b) MDA-MB-468 lysates were treated with 10 μM each compound and incubated at 65°C. Lysates were electrophoretically separated and TP was detected via Western blot. TP bands were normalized to the thermostable SOD1 control. Each treatment was performed once (n = 1).

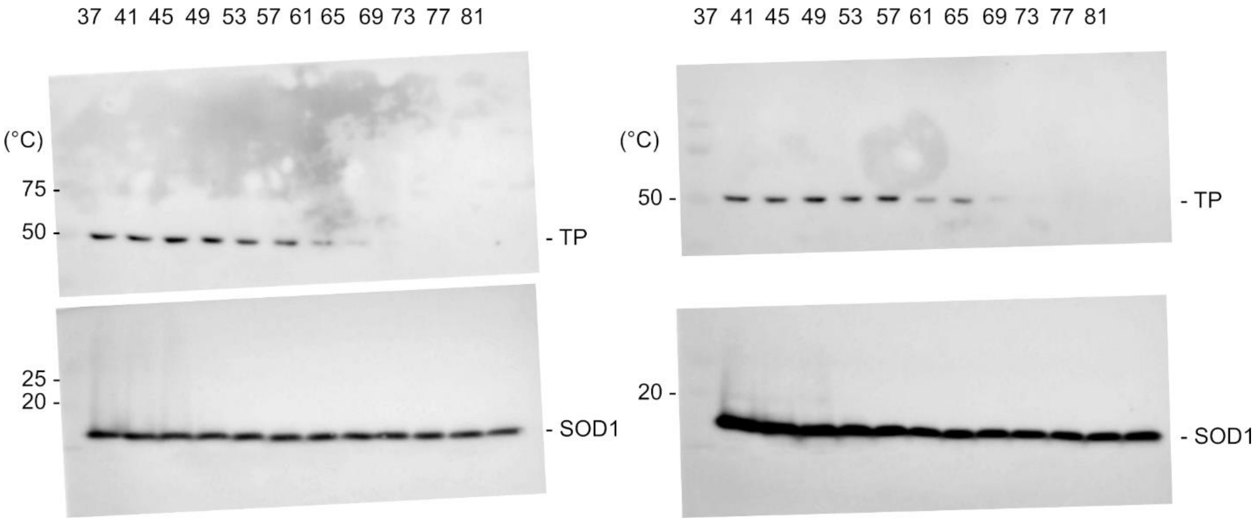

Figure S10. Uncropped Western blots for data presented in **Figure S9a**. MDA-MB-468 lysates were incubated from 37 to 81°C for 3 min. The soluble fraction was separated from insoluble, denatured proteins by centrifugation and separated via SDS-PAGE. anti-TP (from mouse) was used at a dilution of 1:100, anti-SOD1 (from mouse) was used at a dilution of 1:500, and HRP-linked anti-mouse IgG was used at a concentration of 1:3000. The membrane was cut half and TP and SOD1 were imaged separately to aid in achieving better TP signal.

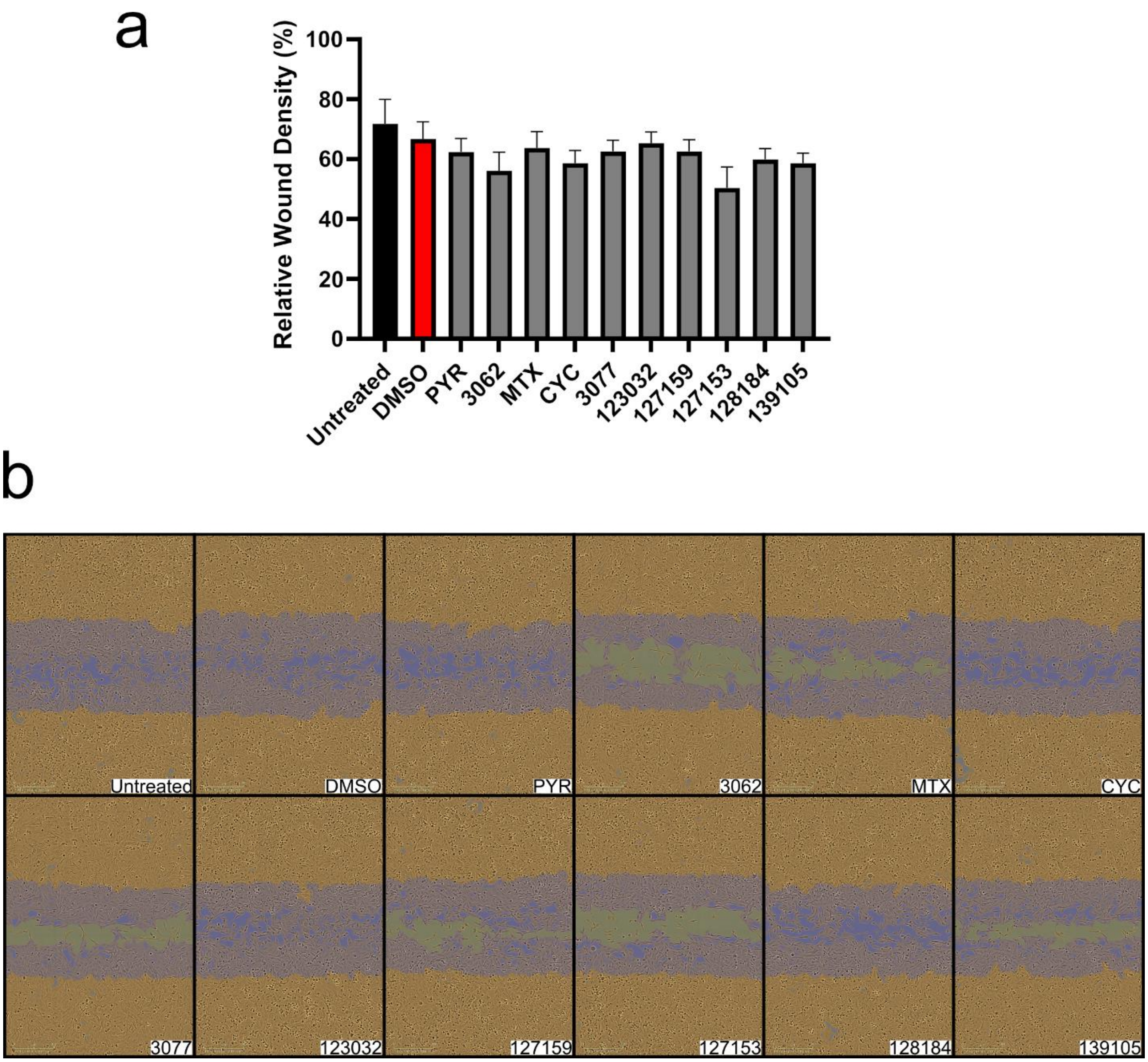

Figure S11. Impact of cycloguanil analogues on wound healing. (a) A wound healing assay was performed using MDA-MB-231 breast cancer cells and each inhibitor at 10 μM for 24 h as treatments. Migration of cells into the wound were monitored using an IncuCyte Zoom microscope. Relative wound densities were compared to the DMSO control (red) using an Ordinary one-way ANOVA with Dunnett's test for multiple comparisons. All comparisons were not significant. Each experiment was performed in quadruplicate and repeated in three separate assays (n = 3). (b) Representative cellular images of each treatment.

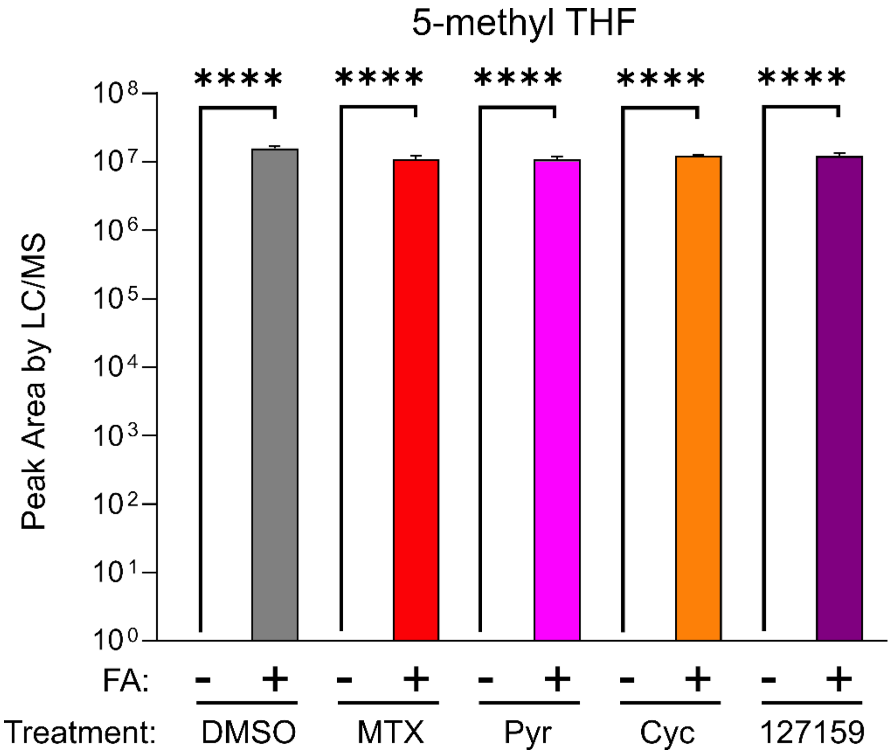

Figure S12. Folinic acid supplementation significantly increases folate species that are downstream of DHFR. An Ordinary one-wave ANOVA with Šídák's correction for multiple comparisons was performed to assess significance. For all treatments, $p < 0.0001$ (****) (n=4).

Table S6. Statistical comparisons for the data presented in **Figure 6**. To determine statistical significance between each treatment alone and DMSO alone and to determine the statistical significance between each treatment supplemented with folinic acid (FA) and DMSO + FA, data were analyzed using an Ordinary one-way ANOVA with Šídák's correction for multiple comparisons. Data are presented separated by analyte (n = 4).

Comparison	Significance	Adjusted *p* value	Comparison	Significance	Adjusted *p* value
UDP					
DMSO *vs* MTX	ns	0.8366	DMSO + FA *vs* MTX + FA	ns	0.9798
DMSO *vs* Pyr	***	0.0004	DMSO + FA *vs* Pyr + FA	ns	0.8180
DMSO *vs* Cyc	***	0.0001	DMSO +FA *vs* Cyc + FA	ns	>0.9999
DMSO *vs* 127159	ns	0.1481	DMSO + FA *vs* 127159 + FA	**	0.0045
orotate					
DMSO *vs* MTX	**	0.0032	DMSO + FA *vs* MTX + FA	ns	0.9995
DMSO *vs* Pyr	ns	0.0696	DMSO + FA *vs* Pyr + FA	ns	0.2753
DMSO *vs* Cyc	ns	0.9755	DMSO +FA *vs* Cyc + FA	ns	0.6586
DMSO *vs* 127159	****	<0.0001	DMSO + FA *vs* 127159 + FA	****	<0.0001
IMP					
DMSO *vs* MTX	****	<0.0001	DMSO + FA *vs* MTX + FA	ns	>0.9999
DMSO *vs* Pyr	****	<0.0001	DMSO + FA *vs* Pyr + FA	**	0.0052
DMSO *vs* Cyc	***	0.0002	DMSO +FA *vs* Cyc + FA	ns	0.9560
DMSO *vs* 127159	****	<0.0001	DMSO + FA *vs* 127159 + FA	**	0.0086
CDP					
DMSO *vs* MTX	****	<0.0001	DMSO + FA *vs* MTX + FA	ns	>0.9999
DMSO *vs* Pyr	****	<0.0001	DMSO + FA *vs* Pyr + FA	ns	0.4617
DMSO *vs* Cyc	****	<0.0001	DMSO +FA *vs* Cyc + FA	ns	>0.9999
DMSO *vs* 127159	****	<0.0001	DMSO + FA *vs* 127159 + FA	****	<0.0001
ADP					
DMSO *vs* MTX	****	<0.0001	DMSO + FA *vs* MTX + FA	**	0.0039
DMSO *vs* Pyr	****	<0.0001	DMSO + FA *vs* Pyr + FA	****	<0.0001
DMSO *vs* Cyc	****	<0.0001	DMSO +FA *vs* Cyc + FA	***	0.0006
DMSO *vs* 127159	****	<0.0001	DMSO + FA *vs* 127159 + FA	****	<0.0001
GDP					
DMSO *vs* MTX	****	<0.0001	DMSO + FA *vs* MTX + FA	ns	>0.9999

DMSO *vs* Pyr	****	<0.0001	DMSO + FA *vs* Pyr + FA	ns	0.6559
DMSO *vs* Cyc	****	<0.0001	DMSO +FA *vs* Cyc + FA	ns	0.9993
DMSO *vs* 127159	****	<0.0001	DMSO + FA *vs* 127159 + FA	*	0.0176
dUMP					
DMSO *vs* MTX	****	<0.0001	DMSO + FA *vs* MTX + FA	ns	>0.9999
DMSO *vs* Pyr	*	0.0244	DMSO + FA *vs* Pyr + FA	ns	>0.9999
DMSO *vs* Cyc	*	0.0321	DMSO +FA *vs* Cyc + FA	ns	>0.9999
DMSO *vs* 127159	****	<0.0001	DMSO + FA *vs* 127159 + FA	ns	>0.9999
GAR					
DMSO *vs* MTX	****	<0.0001	DMSO + FA *vs* MTX + FA	ns	>0.9999
DMSO *vs* Pyr	****	<0.0001	DMSO + FA *vs* Pyr + FA	ns	>0.9999
DMSO *vs* Cyc	****	<0.0001	DMSO +FA *vs* Cyc + FA	ns	>0.9999
DMSO *vs* 127159	****	<0.0001	DMSO + FA *vs* 127159 + FA	***	0.0006
AICAR					
DMSO *vs* MTX	ns	0.7666	DMSO + FA *vs* MTX + FA	ns	0.0782
DMSO *vs* Pyr	****	<0.0001	DMSO + FA *vs* Pyr + FA	ns	>0.9999
DMSO *vs* Cyc	****	<0.0001	DMSO +FA *vs* Cyc + FA	ns	0.6519
DMSO *vs* 127159	*	0.0119	DMSO + FA *vs* 127159 + FA	****	<0.0001

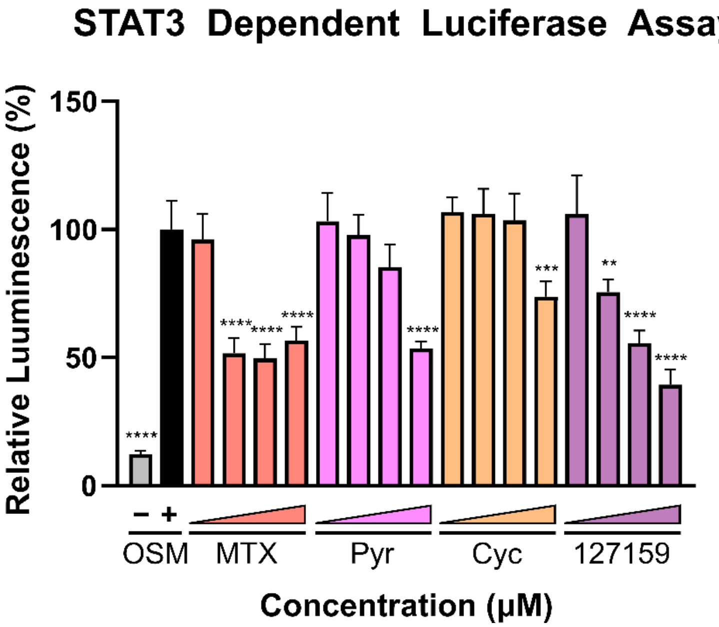

Figure S13. DHFR inhibitors block STAT3-dependent gene expression. U3A cells were incubated with respective inhibitors for 1 h prior to the addition of OSM (10 ng/mL) or vehicle control and incubation for 5 h. Luciferin was added and luminescence measured. Presented data are normalized to the OSM stimulated control (100%), statistical significance was determined using an Ordinary one-way ANOVA with a Dunnett multiple comparison test, (** = $p \leq 0.01$; **** = $p \leq 0.0001$). This is an additional independent experiment complementary to the data presented in **Figure 7** (n=4).

Table S7. Statistical comparisons for the data presented in Figure 7 and Figure S13. To determine statistical significance between each treatment and OSM stimulated cells, data were analyzed using an Ordinary one-way ANOVA with Dunnett's correction for multiple comparisons. Data are presented separated by independent experiment. Technical quadruplicates were performed for each individual experiment.

Treatment	Concentration (μM)	Data from Figure 7		Data from Figure S13	
		Significance	Adjusted *p* value	Significance	Adjusted *p* value
**Unstimulated**	-	****	<0.0001	****	<0.0001
**MTX**	0.02	****	<0.0001	ns	0.9990
	0.2	****	<0.0001	****	<0.0001
	2	****	<0.0001	****	<0.0001
	20	****	<0.0001	****	<0.0001
**Pyr**	0.02	ns	0.4058	ns	0.9992
	0.2	****	<0.0001	ns	0.9995
	2	****	<0.0001	ns	0.1247
	20	****	<0.0001	****	<0.0001
**Cyc**	0.02	****	<0.0001	ns	0.9188
	0.2	****	<0.0001	ns	0.9567
	2	****	<0.0001	ns	0.9991
	20	****	<0.0001	***	0.0004
**127159**	0.02	**	0.0068	ns	0.9672
	0.2	****	<0.0001	**	0.0011
	2	****	<0.0001	****	<0.0001
	20	****	<0.0001	****	<0.0001

Supplementary Methods

Chemical Synthesis of NSC3077

NSC3077 (4,6-diamino-3-(3,4-dichlorophenyl)-2,2-dimethyl-2,3-dihydro-1,3,5-triazine-HCl)

A mixture of 3,4-dichloroaniline (0.309 mmol, 50 mg), 1-cyanoguanidine (0.339 mmol, 28.54 mg), acetone (1.23 mL), and conc. HCl (0.309 mmol, 0.025 mL) was refluxed and stirred for 24 h monitoring reaction completion by TLC. The crude reaction mixture was left at 4 °C overnight yielding crystals that were collected by suction filtration and washed with cold acetone.

NMR data were collected using a Bruker Ascend 400 MHz NMR instrument. ^{1}H NMR, 400 MHz, CD_3OD, δ = 1.50 (d, *J* = 2.8 Hz, 6H, CH_3), 7.39 (d of d, *J* = 2.0 and 8.8 Hz, 1H, CH), 7.69 (d, *J* = 2 Hz, 1H, CH), 7.75 (d, *J* = 8.8 Hz, 1H, CH). ^{13}C NMR (100 MHz, CD_3OD, δ = 27.4, 27.5, 72.0, 131.1, 133.3, 133.4, 135.1, 135.7, 135.9, 158.7, 158.8.

References

1. Heppler, L.N.; Attarha, S.; Persaud, R.; Brown, J.I.; Wang, P.; Petrova, B.; Tošić, I.; Burton, F.B.; Flamand, Y.; Walker, S.R.; et al. The Antimicrobial Drug Pyrimethamine Inhibits STAT3 Transcriptional Activity by Targeting the Enzyme Dihydrofolate Reductase. *J. Biol. Chem.* **2022**, *298*, 101531, doi:10.1016/j.jbc.2021.101531.
